# Electrodeposition of SnO2 on FTO and its Application in Planar Heterojunction Perovskite Solar Cells as an Electron Transport Layer

**DOI:** 10.1186/s11671-017-2247-x

**Published:** 2017-08-16

**Authors:** Yohan Ko, Yeong Rim Kim, Haneol Jang, Chanyong Lee, Man Gu Kang, Yongseok Jun

**Affiliations:** 1 Department of Materials Chemistry and Engineering, Department of Energy Engineering, Konkuk University, Seoul, 143-701 Republic of Korea; 2 IT Materials Technology Research Section, ETRI, Gajeongro 218, Yuseong, Daejeon, Republic of Korea

**Keywords:** SnO_2_, Electrodeposition, Perovskite solar cell, Electrochemistry

## Abstract

We report the performance of perovskite solar cells (PSCs) with an electron transport layer (ETL) consisting of a SnO_2_ thin film obtained by electrochemical deposition. The surface morphology and thickness of the electrodeposited SnO_2_ films were closely related to electrochemical process conditions, i.e., the applied voltage, bath temperature, and deposition time. We investigated the performance of PSCs based on the SnO_2_ films. Remarkably, the experimental factors that are closely associated with the photovoltaic performance were strongly affected by the SnO_2_ ETLs. Finally, to enhance the photovoltaic performance, the surfaces of the SnO_2_ films were modified slightly by TiCl_4_ hydrolysis. This process improves charge extraction and suppresses charge recombination.

**Electronic supplementary material:**

The online version of this article (doi:10.1186/s11671-017-2247-x) contains supplementary material, which is available to authorized users.

## Background

Solar cell devices based on organometallic halide perovskite materials have exhibited unprecedented performance over the brief span of 6 years, and organometallic halide perovskite solar cells (PSCs) show promise as affordable alternative solar cells with high power conversion efficiency (PCE) [1–3]. The huge interest in this new class of solar cells is due to their high absorption coefficient, ambipolar charge transport, small exciton binding energy, and long diffusion length [4–6]. Despite these excellent properties, PSCs possess several drawbacks. The most important of these are the sensitivity of perovskite materials to moisture, heat, and UV irradiation. To address these drawbacks, it has been found that adding formamidinium and/or an inorganic cation (Cs or Rb) to a methylammonium cation improves the stability against these environmental factors [3], and the durability of PSCs thus depends on both the device configuration (n-i-p, p-i-n) and the metal oxide semiconductors [7]. Generally, TiO_2_ materials are widely used in PSCs as electron transport layers (ETLs) in the n-i-p device configuration because of their large band gap and band alignment, and highly efficient PSCs are realized using TiO_2_ ETLs [8]. Although PSCs with TiO_2_ ETLs exhibit remarkable efficiency, the UV sensitivity and electronic properties of TiO_2_ have been suggested as targets for improvement to reduce the hysteresis and obtain durable PSCs [9]. Specifically, Heo et al. reported that Li doping can enhance the carrier mobility and conductivity of TiO_2_ and thus yield PSCs without significant hysteresis [10]. Ito et al. reported that when TiO_2_ in a PSC is exposed to UV irradiation, electrons are extracted at the TiO_2_/perovskite interface, degrading the perovskite material [11].

Stannic oxide (SnO_2_) has been widely studied for diverse applications such as batteries, gas sensors [12], solar cells [13], and catalysts. It is regarded as a promising candidate for use as a transparent conducting material and photoelectrode in photovoltaic devices. Considerable attention has been drawn recently to its application in PSCs as an alternative ETL with the goal of enhancing device performance and light stability, as it has a larger band gap (~3.6 eV at 300 K), higher electrical conductivity, and greater chemical stability than TiO_2_ semiconductors [2]. Various synthetic routes to SnO_2_, including sol–gel methods [14], molten-salt synthesis [15], microwave techniques [16], atomic layer deposition (ALD), and electrochemical deposition (ED) [17–20] have been developed. ALD and spin-coating solution processes are the dominant methods for fabricating SnO_2_ ETLs in PSCs [21–23]. The fabrication of ETLs in photovoltaic devices is paramount for limiting production costs because of the requirements for its production, such as thermal treatment, multiple processing steps, operation control, and scalable processing.

Here, we report on the synthesis and ETL application of SnO_2_ thin films on fluorine-doped tin oxide (FTO) by ED. Among the available methods, electrodeposition has the advantages of reduced production cost and large-scale manufacturing because it does not require a vacuum environment or complex operation control. Considering that perovskite materials are suitable for roll-to-roll manufacturing, the application of electrodeposition to obtain SnO_2_ ETLs will demonstrate not only a simple, cost-effective, and scalable strategy for alternative ETLs but also facilitate development of a continuous roll-to-roll process for industrial application of PSCs.

## Methods

### Preparation of SnO_2_ Film

A chronovoltammetry technique (VSP 200, Biologic) was used for ED of Sn nanospheres onto an FTO substrate using a standard three-electrode system in a deionized water solution (50 mL) containing 0.05 M SnCl_2_∙2H_2_O [tin chloride (Π), Sigma Aldrich] and 1 mL of nitric acid (HNO_3_, Samchun Chemical). The nanospheres were then thermally treated in air at 400 °C for 30 min to obtain SnO_2_. The aqueous solution was stirred for 1 h at 60 °C on a hot plate. After N_2_ purging for 10 min, the solution was used for electrodeposition. In the standard three-electrode system, FTO was used as the working electrode, and a platinum plate was used as the counter electrode. The reference electrode was a Ag/AgCl electrode (CHI111) in 1 M KCl solution.

### Device Fabrication

The prepared SnO_2_ thin films on FTO (TEC 8) were used in the fabrication of PSCs. The perovskite layer was processed in two steps. A mixture of PbI_2_ (99.999%, Aldrich) and PbCl_2_ (99.999%, Aldrich) was dissolved in *N*, *N*-dimethylformamide and stirred at 60 °C. The molar ratio of the precursor solution (PbI_2_:PbCl_2_) was 1:1 (1 M). The PbI_2_/ PbCl_2_ solution was spin-coated on the SnO_2_-coated FTO at 5000 rpm for 30 s in a glove box and dried on a hotplate at 70 °C. To convert it to a perovskite material, 120 μL of methylammonium iodide solution (40 mg/mL) was loaded at 0 rpm for 35 s and then spin-coated at 3500 rpm for 20 s; the sample was then annealed isothermally at 105 °C for 75 min in the ambient environment. After annealing, the films were moved into the glove box in N_2_ atmosphere, and a hole-transporting material (HTM) was spin-coated on the MAPbI_3-x_Cl_x_/SnO_2_/FTO film at 3000 rpm for 30 s. Poly[bis(4-phenyl)(2,4,6-trimethylphenyl)amine] (EM Index) solution (20 mg/1 mL) was used as the HTM with 15 μL of Li-bis(trifluoromethanesulfonyl)imide)/acetonitrile (170 mg/1 mL) and 15 μL of tert-butylpyrridine. Finally, Au was deposited via thermal evaporation. TiCl_4_ hydrolysis treatment was applied by immersing the electrodeposited SnO_2_ films in a 40 mM TiCl_4_ solution at 70 °C for 30 min and drying them at 150 °C in air.

### Characterization

Cyclic voltammetry (CV, scan rate 50 mV/s) measurements were made to confirm the electrochemical behavior of the SnCl_2_∙2H_2_O solution from −1.5 to 2 V. The crystalline structure of the samples was characterized by X-ray diffraction (XRD, Rigaku, Dmax 2200, Cu Kα) and X-ray photoelectron spectroscopy (XPS, ULVAC-PHI 5000, VersaProbe II). The morphologies of the samples were observed by field emission scanning electron microscopy (SEM, Hitachi S4800). The *J*–*V* curves of the PSCs were obtained using an electrochemical station (VSP200, Bio-Logic) under 100 mW/cm^2^ AM 1.5G light (Sun 3000 class AAA, ABET Technology) with a metal mask 0.098 cm^2^ in area. Devices were scanned at a 20 mV/s scan rate. CV measurements of the blocking layer effect were performed using a three-electrode setup after nitrogen purging for 10 min. The aqueous electrolyte contained 0.5 M KCl and the electron redox couple K_4_[Fe(II)(CN)_6_]/K_3_[Fe(III)(CN)_6_] at a concentration of 5 mM. A Ag/AgCl electrode was used for the reference electrode, and a Pt wire was used for the counter electrode; the scan rate was 50 mV/s. An Oriel-calibrated Si solar cell (SRC-1000-TC-KG5-N) was used to adjust the light intensity to one-sun illumination. The external quantum efficiency (EQE) was measured using an Ivium potentiostat and a monochromator (DongWoo Optron Co., Ltd.) under a light support (ABET 150 W xenon lamp, ABET Technology). EQE data were acquired in DC mode. Photoluminescence (PL) spectra were measured using a luminescence spectrometer (LS 55, PerkinElmer) with excitation at 530 nm. The intensity-modulated photocurrent and photovoltage were measured by an Ivium potentiostat with a Modulight LED (Ivium).

## Results and Discussion

**Fig. 1:**
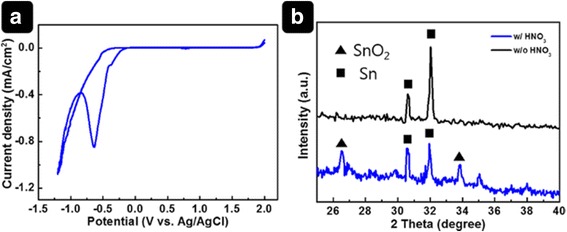
(**a**) CV curve measured at a scan rate of 50 mV/s and (**b**) XRD patterns of electrodeposited SnO_2_

We performed CV measurements of the SnCl_2_∙2H_2_O solution to identify suitable potential values. Figure 1a shows the CV curve, which was scanned from 2.0 to −1.2 V. All potential values were recorded with respect to the reference electrode (Ag/AgCl). As shown in Fig. 1a, an increase in the cathodic current was observed from −0.5 to −1.2 V. Generally, when the voltage is swept in a CV experiment from positive to negative voltage, the current first increases because of an electrochemical reaction on the working electrode surface and then decreases owing to local depletion of the chemical species close to the working electrode.

**Fig. 2:**
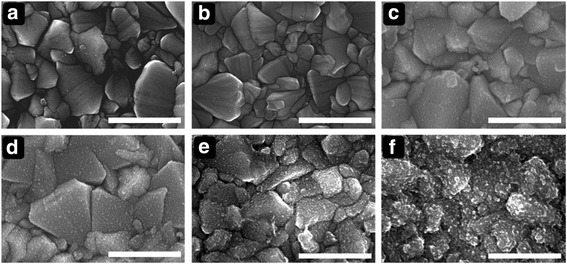
Top-view SEM images of SnO_2_ films electrodeposited at various applied voltages. (**a**) − 0.5 V, (**b**) −0.6 V, (**c**) −0.7 V, (**d**) −0.8 V, (**e**) −0.9 V, and (**f**) −1.0 V vs. Ag/AgCl. *Scale bar* is 1 μm

On the basis of the CV result, we performed ED using a chronovoltammetry technique. Note that the phase of the deposits depends on the concentration ratio of [HNO_3_] to [Sn^2+^] because nitric acid acts as an oxygen source in the phase [24]. The presence of HNO_3_ (as identified in the XRD pattern, Fig. 1b) facilitated generation of a SnO_2_–Sn co-phase. This will be referred to as SnO_2_–Sn nanospheres to distinguish it from pure SnO_2_. Figure 2 shows SEM images of the SnO_2_–Sn nanospheres deposited on FTO substrates at different potential values (−0.5, −0.6, −0.7, −0.8, −0.9, and −1 V). We found that the applied voltage is a very important parameter in the electrodeposition process, as the morphologies of the deposits were dramatically different. For relatively low absolute potentials (−0.5 and −0.6 V), few SnO_2_–Sn nanospheres formed. On the other hand, the FTO was overlaid with Sn having irregular shapes at −0.9 and −1 V. Even though comparable SnO_2_–Sn nanosphere formation occurred at −0.7 and −0.8 V, the uniformity was better at −0.7 V. As a result of these observations, −0.7 V was chosen as a suitable potential for electrodeposition of SnO_2_–Sn nanospheres.

**Fig. 3:**
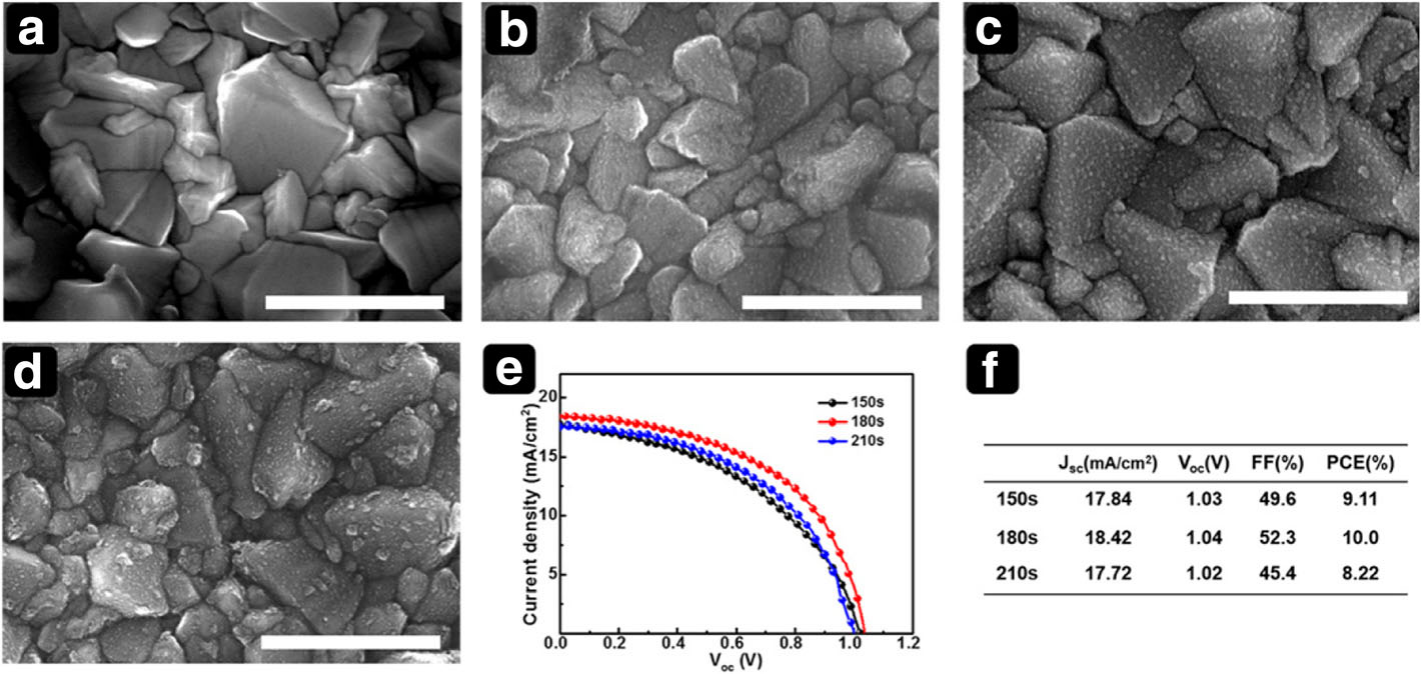
Top-view SEM images of substrates for different deposition time. (**a**) Bare FTO and SnO_2_ films deposited for (**b**) 150 s, (**c**) 180 s, and (**d**) 210 s. Corresponding photovoltaic performance: (**e**) *J*–*V* curves and (**f**) photovoltaic parameters of PSCs with electrodeposited SnO_2_ ETL. *Scale bar* is 1 μm

A potential of −0.7 V was also used to optimize the deposition time in the range of 150 to 210 s. Figure 3 shows SEM images of samples obtained at various deposition times and the corresponding device performance. Fewer particles formed at 150 s than at 180 s. For a longer deposition time (210 s), aggregation of SnO_2_–Sn nanospheres was confirmed. To evaluate the photovoltaic performance of PSCs with the electrodeposited SnO_2_ films, the SnO_2_–Sn nanosphere films were thermally treated in air at 450 °C for 30 min to obtain fully converted SnO_2_ films. A CH_3_NH_3_PbI_3-x_Cl_x_ perovskite layer was fabricated via a PbICl-seed-layer-assisted interdiffusion process. Details are provided in the experimental section. As shown in Fig. 3e, f, for a deposition time of 150 s, the short-circuit current density (*J*_sc_), open-circuit voltage (*V*_oc_), fill factor (FF), and PCE (%) were 17.84 mA/cm^2^, 1.03, 0.496, and 9.11%, respectively. As the deposition time increased from 150 to 180 s, *J*_sc_ improved, and a higher PCE of 10.0 was obtained. The use of a deposition time of 210 s mainly affected the *J*_sc_ and FF value, leading to a lower PCE of 8.22. To gain further insight into parasitic resistances, we calculated series resistance (*R*_s_) and shunt resistance (*R*_sh_) from the *J–V* curves. *R*_s_ values are 10.4, 5.2, and 12.5 (ohm cm^2^); *R*_sh_ values are 194.9, 558.5, and 167.1 (ohm cm^2^) for the time of 150, 180, and 210 s, respectively. The calculated parasitic resistances explain device performances in operation obtained from different electrochemical deposition condition. As shown in the SEM image in Fig. 3d, the poor morphology of the SnO_2_ film at a deposition time of 210 s is expected to impede charge transfer between CH_3_NH_3_PbI_3-x_Cl_x_ and FTO, resulting in a reduced *J*_sc_.

**Fig. 4:**
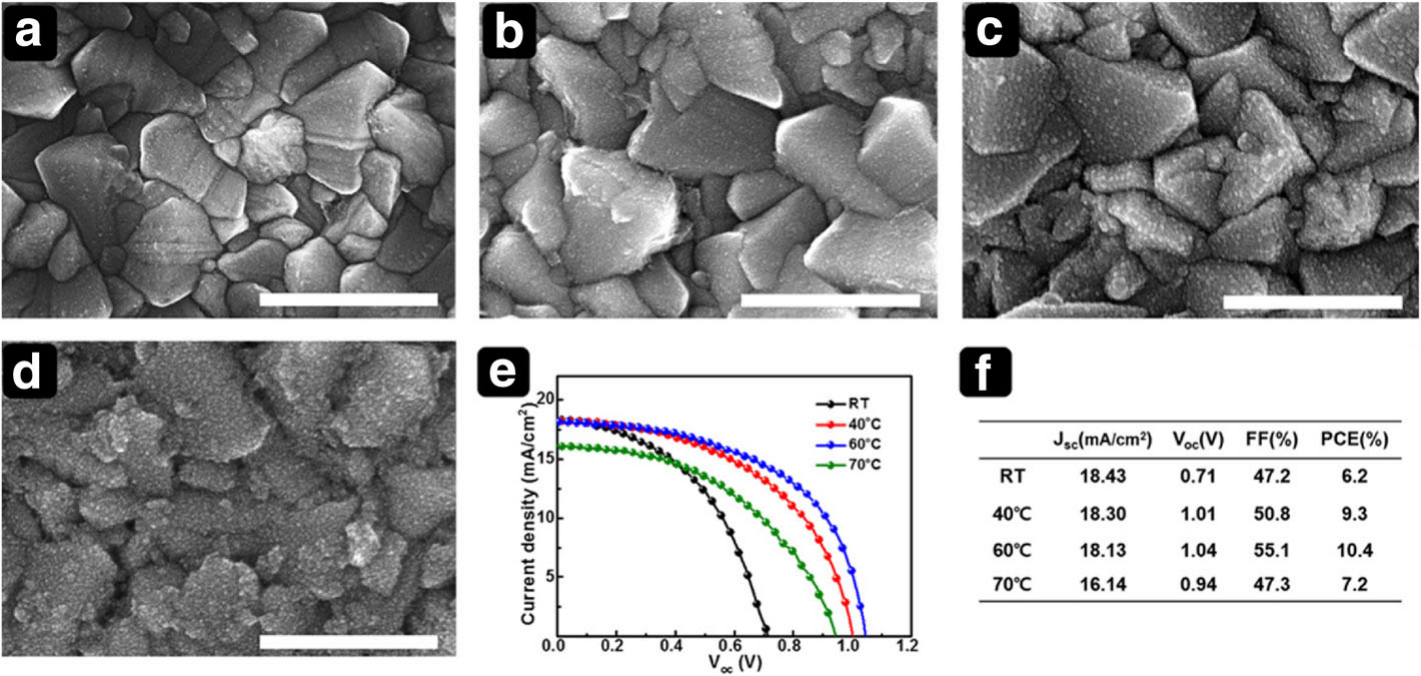
Top-view SEM images of SnO_2_ films electrodeposited at various bath temperatures. (**a**) RT, (**b**) 40 °C, e(**c**) 60 °C, and (**d**) 70 °C. Corresponding photovoltaic performance: (**e**) *J*–*V* curves and (**f**) photovoltaic parameters of PSCs with electrodeposited SnO_2_ ETL. *Scale bar* is 1 μm

Considering that the electrodeposition process depends on the ion mobility in an electrolyte solution, we also explored the effect of temperature on the morphology of the films. Figure 4 shows top-view SEM images of films deposited at different bath temperatures with −0.7 V for 180 s. As expected, the surface morphology of the SnO_2_–Sn nanospheres prepared at different bath temperatures varies. The nanosphere size, roughness, and thickness seem to be affected, as the migration of Sn^2+^ ions was enhanced at higher temperature. The photovoltaic efficiency of PSCs fabricated using these films is compared in Fig. 4e, f. A finer SnO_2_ film yields better performance, and the optimum efficiency was obtained for the film deposited at 60 °C. The SnO_2_ film morphology is expected to significantly affect the PSC performance because planar PSCs have a direct interface between the ETL and the perovskite layer. The improved conformality could result in good contact that affords enhanced electron transport [25]. The SEM images of perovskite layer fabricated from varied ETLs were provided in supporting information (SI) Additional file 1: Figure S1.

**Fig. 5:**
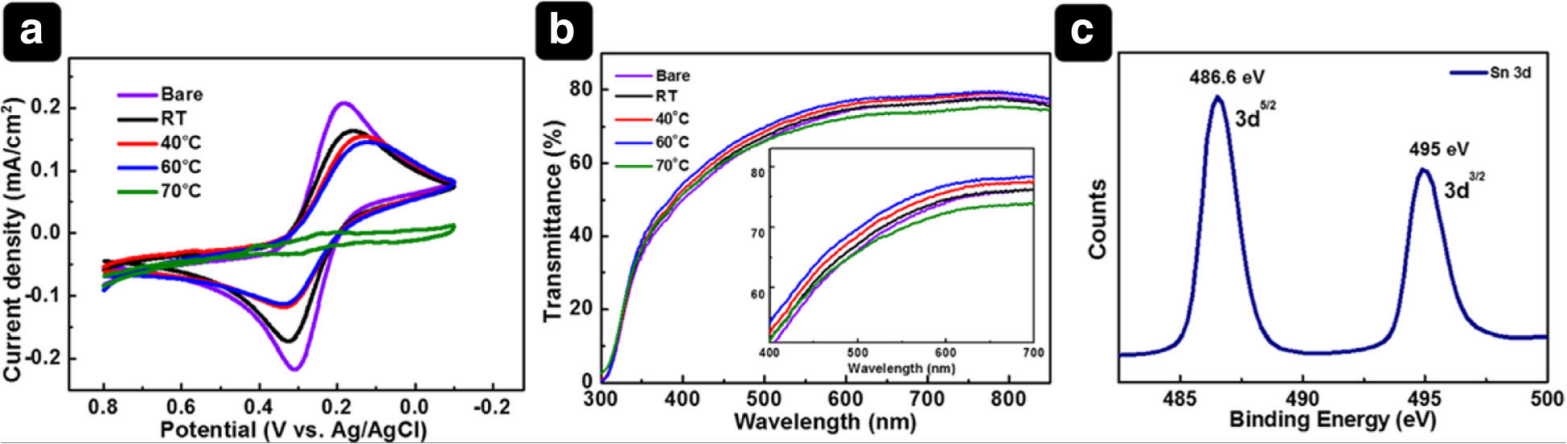
Various analyses for the films. (**a**) CV curves in redox solution system and (**b**) transmission spectra of bare FTO and SnO_2_ films electrodeposited at different bath temperatures in redox solution system. (**c**) XPS Sn 3d spectrum of thermally treated SnO_2_ film

To further examine the effect of temperature on the morphology with respect to the blocking effect of the electrodeposited SnO_2_ films, we conducted CV measurements in an aqueous electrolyte containing [Fe(CN)_6_]^3−^/[Fe(CN)_6_]^4−^ because the redox reaction depends on charge transfer between the FTO and the electrolyte [26]. The electron transfer kinetics can be interpreted by extracting the separation of the peak potentials and peak current of a redox system from the CV curves. If the redox reaction between [Fe(CN)_6_]^3−^/[Fe(CN)_6_]^4−^ ions is hampered by the SnO_2_ layer, the oxidized and reduced forms of the redox couple exhibit peak potentials that are shifted away from the control on bare FTO and become semireversible; consequently, the peak current density will be reduced [27]. Figure 5a shows the CV curves of bare FTO and the SnO_2_ films. The CV curve of bare FTO clearly shows a reversible redox reaction, indicating a lower barrier to electron transfer. In contrast, the FTO with electrodeposited SnO_2_ exhibits a larger peak-to-peak separation (Δ*E*_p_) of the cathodic and anodic peak potentials compared to that of bare FTO. The Δ*E*_p_ values of films deposited at room temperature (RT), 40, 60, and 70 °C are 125, 175, 207, and 230 mV, respectively. This indicates that the kinetics of the redox reaction are changed by the blocking effect of the SnO_2_ films. In contrast, charge transfer at the FTO is highly suppressed by the film deposited at 70 °C, implying that the SnO_2_ is densely deposited onto the FTO. The thick SnO_2_ film could result in less effective and slower electron transport, negatively affecting the photovoltaic performance. The cathodic peak current (*I*_p_) of the films decreased with increasing bath temperature, indicating that the FTO coverage was improved.

On the basis of the CV results and SEM images, we could speculate that the FTO electrode at low temperature is covered with fewer nanoparticles; therefore, we conclude that the SnO_2_ film fabricated at 60 °C has a suitable thickness and morphology for use in PSCs and has a dominant effect on the device performance. The optical transmission of the SnO_2_ films is also compared (Fig. 5b). As the bath temperature increases from RT to 60 °C, the transmittance of the SnO_2_ films is enhanced compared to that of FTO. At a high bath temperature of 70 °C, the transmittance is inferior to that of FTO, which is attributed to the increased film thickness, as evidenced by the SEM image.

XPS was performed to measure the composition of the electrodeposited films. The XPS spectrum of the thermally treated SnO_2_ film is shown in Fig. 5c. Sn 3d_5/2_ and Sn 3d_3/2_ peaks at binding energies of 486.6 and 495 eV, respectively, were observed, whereas the film without heat treatment showed Sn 3d_5/2_ and Sn 3d_3/2_ peaks at 484.8 and 493.2 eV, respectively (SI, Additional file 1: Figure S2) [21]. The SnO_2_ film is clearly obtained through heat treatment.

**Fig. 6:**
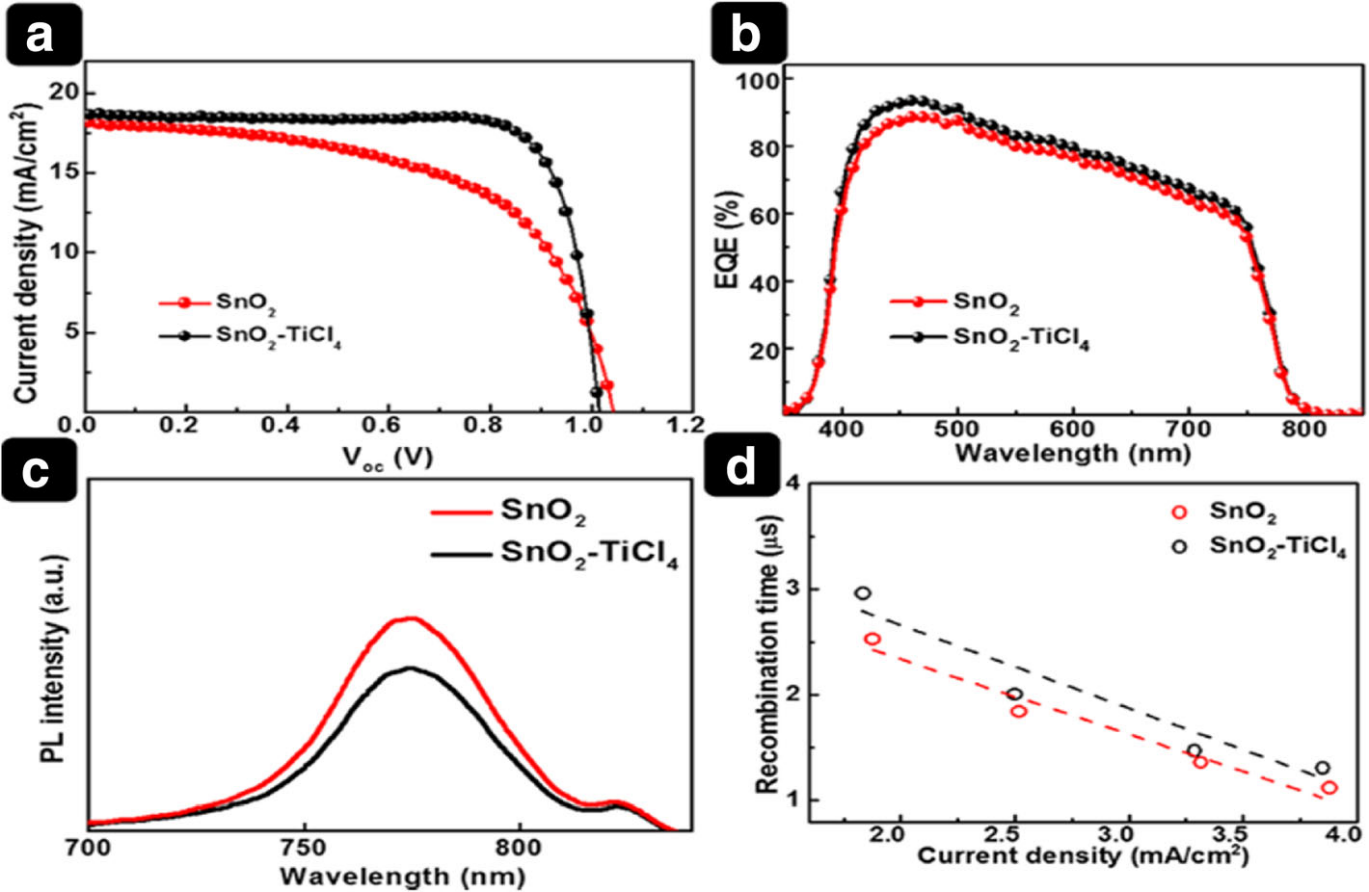
Cell performance with IPCE and PL data. (**a**) *J*–*V* curves and (**b**) EQE spectra of PSC devices based on SnO_2_ and SnO_2_–TiCl_4_. (**c**) Steady-state PL spectra of FTO/SnO_2_/perovskite and FTO/SnO_2_-TiCl_4_/perovskite samples. (**d**) Recombination time versus current density

On the other hand, although SnO_2_ electrodeposition provides a versatile and low-cost route toward scalable manufacturing systems [28], the demonstrated photovoltaic performance of the electrodeposited SnO_2_ films is not impressive. To improve the device performance, TiCl_4_ treatment was used to modify the SnO_2_ surface. As shown in Fig. 6a, the device based on SnO_2_ without TiCl_4_ treatment shows a *J*_sc_ value of 18.12 mA/cm^2^, a *V*_oc_ value of 1.04 V, a FF of 57.3%, and a PCE of 10.83%. In comparison, the device based on SnO_2_ with TiCl_4_ treatment (SnO_2_–TiCl_4_) exhibits a *J*_sc_ value of 18.65 mA/cm^2^, a *V*_oc_ value of 1.02 V, a FF of 79.1%, and a PCE of 14.97% (a 38% enhancement). The efficiency improvement is attributed mainly to the improved *J*_sc_ and FF.

To understand the mechanism by which TiCl_4_ treatment improves the *J*_sc_ value, we measured the EQE (Fig. 6b). The EQE of the SnO_2_–TiCl_4_ device shows an increase from 17.8 to 18.6 mA/cm^2^ in the entire wavelength spectral region. The enhancement in the EQE after TiCl_4_ treatment is in good agreement with the improved *J*_sc_ in the *J*–*V* curves, which implies efficient charge collection. The EQE enhancement is expected to be originated from a better injection of electrons at the ETLs/perovskite interface [29, 30]. To further investigate the electron injection, the steady-state PL was measured for substrates with both ETLs. Figure 6c shows the PL spectra of the FTO/SnO_2_/perovskite and FTO/SnO_2_–TiCl_4_/perovskite samples. Compared to the SnO_2_-based film, the SnO_2_–TiCl_4_-based film exhibited reduced PL intensity, indicating that electron transfer from the perovskite to the ETL was enhanced by TiCl_4_ treatment since the PL emission of perovskite layer is quenched by contact. Possibly, the enhanced electron injection in ETLs with TiCl_4_ treatment improved the EQE. To further examine the improved performance of the SnO_2_–TiCl_4_-based device, intensity-modulated photovoltage spectroscopy (IMVS, Additional file 1: Figure S3) was performed to characterize the recombination time (*τ*_r_) (Fig. 6d). The recombination lifetime depends on the concentration of charge carriers in the solar cell. Thus, the recombination time is influenced by the current density, which is modulated by varying the light intensity. The carrier recombination time for the SnO_2_–TiCl_4_-based device was 1.17 times longer than that of the SnO_2_-based devices. The longer time constant for recombination is expected to afford an increase in *J*_sc_, FF, and better device performance [31, 32]. The device statistics (30 samples for each) were provided in Additional file 1: Figure S4.

## Conclusions

In summary, we demonstrated a versatile and scalable electrodeposition technique to obtain a SnO_2_ ETL for planar heterojunction PSCs. The properties of the electrodeposited SnO_2_ depended strongly on the deposition time, electrolyte bath temperature, and applied voltage. Moreover, devices based on SnO_2_ treated with TiCl_4_ showed significantly enhanced *V*_oc_ and *J*_sc_, leading to a PCE enhancement of 42%.

## Additional file

Additional file 1: Figure S1. SEM images of perovskite layer prepared from varied ETLs. (a) RT of SnO_2_, (b) 40°C of SnO_2_, (c) 60°C of SnO_2_, (d) 70°C of SnO_2_, and (e) 60°C of SnO_2_-TiCl_4._ Figure S2 XPS spectrum of SnO_2_ film without thermal treatment. Figure S3 IMVS curves of (a) SnO_2_-based and (b) SnO_2_–TiCl_4_-based devices. Figure S4 Performance statistic of devices based on different ETLs. (DOCX 3379 kb)

## Abbreviations

ALD: Atomic layer deposition
CV: Cyclic voltammetry
ED: Electrochemical deposition
EQE: External quantum efficiency
ETL: Electron transport layer
FF: Fill factor
FTO: Fluorine-doped tin oxide
HTM: Hole-transporting material
IMVS: Intensity-modulated photovoltage spectroscopy
PCE: Power conversion efficiency
PL: Photoluminescence
PSC: Perovskite solar cell
RT: Room temperature
SEM: Scanning electron microscopy
XPS: X-ray photoelectron spectroscopy
XRD: X-ray diffraction

## References

[1] Kojima A, Teshima K, Shirai Y, Miyasaka T (2009). Organometal halide perovskites as visible-light sensitizers for photovoltaic cells. J Am Chem Soc.

[2] Ke W, Fang G, Liu Q, Xiong L, Qin P, Tao H, Wang J, Lei H, Li B, Wan J, Yang G, Yan Y (2015). Low-temperature solution-processed tin oxide as an alternative electron transporting layer for efficient perovskite solar cells. J Am Chem Soc.

[3] Saliba M, Matsui T, Domanski K, Seo J-Y, Ummadisingu A, Zakeeruddin SM, Correa-Baena J-P, Tress WR, Abate A, Hagfeldt A, Grätzel M (2017) Incorporation of rubidium cations into perovskite solar cells improves photovoltaic performance. doi:10.1126/science.aah5557 10.1126/science.aah5557 27708053

[4] Miyata A, Mitioglu A, Plochocka P, Portugall O, Wang JT-W, Stranks SD, Snaith HJ, Nicholas RJ (2015). Direct measurement of the exciton binding energy and effective masses for charge carriers in organic–inorganic tri-halide perovskites. Nat Phys.

[5] Xing G, Mathews N, Sun S, Lim SS, Lam YM, Grätzel M, Mhaisalkar S, Sum TC (2015) Long-range balanced electron and hole-transport lengths in organic-inorganic MAPbI3. Science 324. doi:10.1126/science.1243167 10.1126/science.1243167 24136965

[6] Stranks SD, Eperon GE, Grancini G, Menelaou C, MJP A, Leijtens T, Herz LM, Petrozza A, Snaith HJ (2013). Electron-hole diffusion lengths exceeding 1 micrometer in an organometal trihalide perovskite absorber. Science.

[7] Meng L, You J, Guo TF, Yang Y (2016). Recent advances in the inverted planar structure of perovskite solar cells. Acc Chem Res.

[8] Park NG (2016). Methodologies for high efficiency perovskite solar cells. Nano Convergence.

[9] Jeon NJ, Noh JH, Kim YC, Yang WS, Ryu S, Seok SI (2014). Solvent engineering for high-performance inorganic-organic hybrid perovskite solar cells. Nat Mater.

[10] Heo JH, You MS, Chang MH, Yin W, Ahn TK, Lee S-J, Sung S-J, Kim DH, Im SH (2015). Hysteresis-less mesoscopic CH3NH3PbI3 perovskite hybrid solar cells by introduction of Li-treated TiO2 electrode. Nano Energy.

[11] Ito S, Tanaka S, Manabe K, Nishino H (2014). Effects of surface blocking layer of Sb2S3on nanocrystalline TiO2for CH3NH3PbI3Perovskite solar cells. J Phys Chem C.

[12] Hui-Chi Chiu C-SY (2007). Hydrothermal synthesis of SnO2 nanoparticles and their gas-sensing of alcohol. J Phys Chem.

[13] Yasuhiro Tachibana KH, Takano S, Sayama K, Arakawa H (2002). Investigations on anodic photocurrent loss processes in dye sensitized solar cells: comparison between nanocrystalline SnO2 and TiO2 films. Chem Phys Lett.

[14] Aziz M, Abbas SS, Baharom WRW, Mahmud WZW (2012). Structure of SnO2 nanoparticles by sol–gel method. Mater Lett.

[15] Wang D, Chu X, Gong M (2006). Gas-sensing properties of sensors based on single-crystalline SnO2 nanorods prepared by a simple molten-salt method. Sensors Actuators B Chem.

[16] Krishnakumar T, Jayaprakash R, Parthibavarman M, Phani AR, Singh VN, Mehta BR (2009). Microwave-assisted synthesis and investigation of SnO2 nanoparticles. Mater Lett.

[17] Chen Z, Tian Y, Li S, Zheng H, Zhang W (2012). Electrodeposition of arborous structure nanocrystalline SnO2 and application in flexible dye-sensitized solar cells. J Alloys Compd.

[18] Lee K-T, Lu S-Y (2012). Porous FTO thin layers created with a facile one-step Sn4+−based anodic deposition process and their potential applications in ion sensing. J Mater Chem.

[19] Lee K-T, Lu S-Y (2013). One-step Sn4+−based anodic deposition for flattening of fluorine-doped tin oxide enabling large transmittance enhancements. RSC Adv.

[20] Chu D, Masuda Y, Ohji T, Kato K (2011). Fast synthesis, optical and bio-sensor properties of SnO2 nanostructures by electrochemical deposition. Chem Eng J.

[21] Ren X, Yang D, Yang Z, Feng J, Zhu X, Niu J, Liu Y, Zhao W, Liu SF (2017). Solution-processed Nb: SnO2 electron transport layer for efficient planar perovskite solar cells. ACS Appl Mater Interfaces.

[22] Correa Baena JP, Steier L, Tress W, Saliba M, Neutzner S, Matsui T, Giordano F, Jacobsson TJ, Srimath Kandada AR, Zakeeruddin SM, Petrozza A, Abate A, Nazeeruddin MK, Grätzel M, Hagfeldt A (2015). Highly efficient planar perovskite solar cells through band alignment engineering. Energy Environ Sci.

[23] Chen H, Liu D, Wang Y, Wang C, Zhang T, Zhang P, Sarvari H, Chen Z, Li S (2017). Enhanced performance of planar perovskite solar cells using low-temperature solution-processed al-doped SnO2 as electron transport layers. Nanoscale Res Lett.

[24] Chen X, Liang J, Zhou Z, Duan H, Li B, Yang Q (2010). The preparation of SnO2 film by electrodeposition. Mater Res Bull.

[25] Roelofs KE, Pool VL, Bobb-Semple DA, Palmstrom AF, Santra PK, Van Campen DG, Toney MF, Bent SF (2016). Impact of conformality and crystallinity for ultrathin 4 nm compact TiO2 layers in perovskite solar cells. Adv Mater Interfaces.

[26] Moehl T, Im JH, Lee YH, Domanski K, Giordano F, Zakeeruddin SM, Dar MI, Heiniger LP, Nazeeruddin MK, Park NG, Gratzel M (2014). Strong photocurrent amplification in perovskite solar cells with a porous TiO2 blocking layer under reverse bias. J Phys Chem Letters.

[27] Harnisch F, Freguia S (2012). A basic tutorial on cyclic voltammetry for the investigation of electroactive microbial biofilms. Chem Asian J.

[28] Vequizo JJM, Wang J, Ichimura M (2010). Electrodeposition of SnO2Thin films from aqueous tin sulfate solutions. Jpn J Appl Phys.

[29] Liu D, Li S, Zhang P, Wang Y, Zhang R, Sarvari H, Wang F, Wu J, Wang Z, Chen ZD (2017). Efficient planar heterojunction perovskite solar cells with Li-doped compact TiO 2 layer. Nano Energy.

[30] Agresti A, Pescetelli S, Cinà L, Konios D, Kakavelakis G, Kymakis E, Carlo AD (2016). Efficiency and stability enhancement in perovskite solar cells by inserting lithium-neutralized graphene oxide as electron transporting layer. Adv Funct Mater.

[31] Li S, Zhang P, Wang Y, Sarvari H, Liu D, Wu J, Yang Y, Wang Z, Chen ZD (2016). Interface engineering of high efficiency perovskite solar cells based on ZnO nanorods using atomic layer deposition. Nano Res.

[32] Li S, Zhang P, Chen H, Wang Y, Liu D, Wu J, Sarvari H, Chen ZD (2017). Mesoporous PbI2 assisted growth of large perovskite grains for efficient perovskite solar cells based on ZnO nanorods. J Power Sources.

