# Supplementary material for: Electrodeposition of SnO2 on FTO and its Application in Planar Heterojunction Perovskite Solar Cells as an Electron Transport Layer
Source: Nanoscale Res Lett. 2017 Aug 16;12:498. doi: 10.1186/s11671-017-2247-x (PMC5559410; doi:10.1186/s11671-017-2247-x)
Supplement: Additional file 1: Figure S1. — SEM images of perovskite layer prepared from varied ETLs. (a) RT of SnO2, (b) 40°C of SnO2, (c) 60°C of SnO2, (d) 70°C of SnO2, and (e) 60°C of SnO2-TiCl4. Figure S2 XPS spectrum of SnO2 film without thermal treatment. Figure S3 IMVS curves of (a) SnO2-based and (b) SnO2–TiCl4-based devices. Figure S4 Performance statistic of devices based on different ETLs. (DOCX 3379 kb) [file 11671_2017_2247_MOESM1_ESM.docx]

**Supporting information, figures**


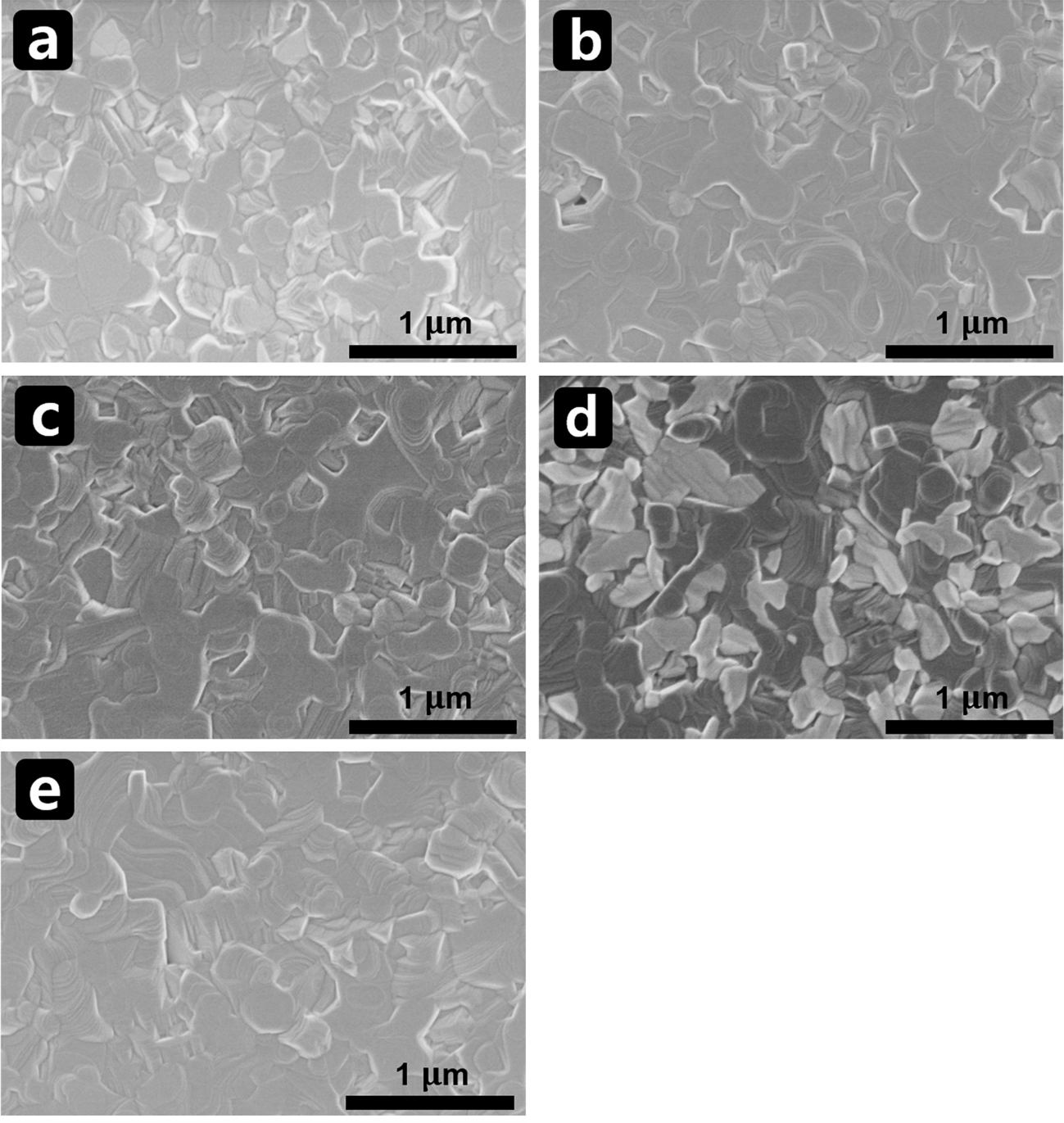
Figure S1

**
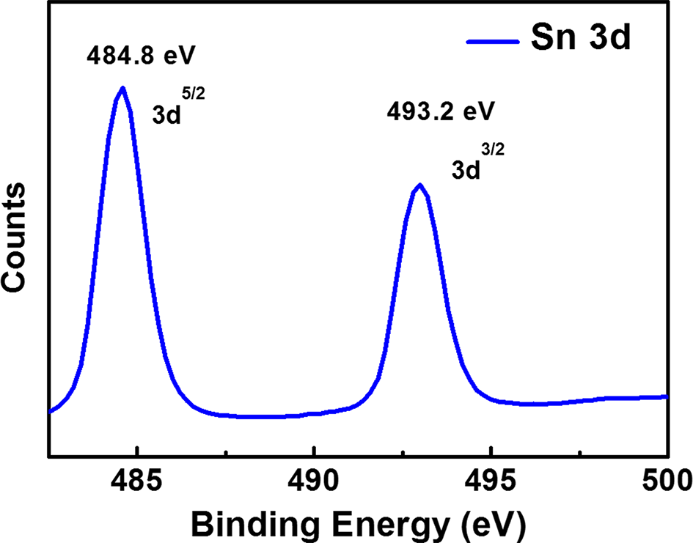
**Figure S2

**
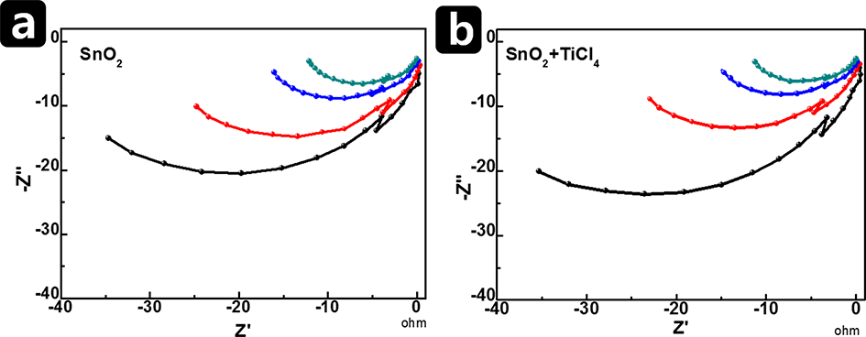
**Figure S3


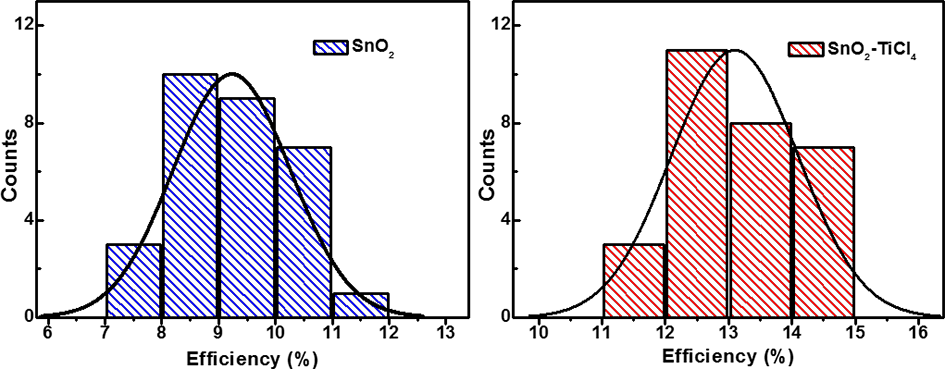
Figure S4

**Figure captions for the Supporting information**

Fig. S1 SEM images of perovskite layer prepared from varied ETLs. (a) RT of SnO_2_, (b) 40°C of SnO_2_, (c) 60°C of SnO_2_, (d) 70°C of SnO_2_ and (e) 60°C of SnO_2_ - TiCl_4_

Fig. S2 XPS spectrum of SnO_2_ film without thermal treatment

Fig. S3 IMVS curves of (a) SnO_2_-based and (b) SnO_2_–TiCl_4_-based devices

Fig. S4 Performance statistic of devices based on different ETLs
